# *M tuberculosis* in the Adjuvant Modulates Time of Appearance of CNS-Specific Effector T Cells in the Spleen through a Polymorphic Site of TLR2

**DOI:** 10.1371/journal.pone.0055819

**Published:** 2013-02-11

**Authors:** Chiara Nicolò, Gabriele Di Sante, Annabella Procoli, Giuseppe Migliara, Alessia Piermattei, Mariagrazia Valentini, Giovanni Delogu, Achille Cittadini, Gabriela Constantin, Francesco Ria

**Affiliations:** 1 Institute of General Pathology, Università Cattolica del S. Cuore, Rome, Italy; 2 Institute of Microbiology, Università Cattolica del S. Cuore, Rome, Italy; 3 Institute of Gynecology, Università Cattolica del S. Cuore, Rome, Italy; 4 Section of General Pathology, Department of Pathology and Diagnostics, University of Verona, Verona, Italy; International Center for Genetic Engineering and Biotechnology, India

## Abstract

DC deliver information regulating trafficking of effector T cells along T-cell priming. However, the role of pathogen-derived motives in the regulation of movement of T cells has not been studied. We hereinafter report that amount of *M tuberculosis* in the adjuvant modulates relocation of PLP139-151 specific T cells. In the presence of a low dose of *M tuberculosis* in the adjuvant, T cells (detected by CDR3 BV-BJ spectratyping, the so-called “immunoscope”) mostly reach the spleen by day 28 after immunization (“late relocation”) in the SJL strain, whereas T cells reach the spleen by d 14 with a high dose of *M tuberculosis* (“early relocation”). The C57Bl/6 background confers a dominant “early relocation” phenotype to F1 (SJL×C57Bl/6) mice, allowing early relocation of T cells in the presence of low dose *M tuberculosis*. A single non-synonymous polymorphism of TLR2 is responsible for “early/late” relocation phenotype. Egress of T lymphocytes is regulated by TLR2 expressed on T cells. Thus, pathogens engaging TLR2 on T cells regulate directly T-cell trafficking, and polymorphisms of TLR2 condition T-cell trafficking upon a limiting concentration of ligand.

## Introduction

Trafficking of effector T cells plays a key role in the response to pathogens, immunosurveillance and autoimmunity. T-cell activation is accompanied by a modification of the cell surface asset of several molecules involved in cell adhesion and migration that are consequently differentially expressed on naïve, effector and memory T cells [1].

The ability of cells of the innate immune system to “sense” pathogen-related moieties relies on Pattern-recognition receptors (PRRs). Most PRRs are polymorphic in humans and mice, and these polymorphisms bear differences in the control of infectious pathogens [2]. DC express most, if not all, of the PRRs. Following engagement of PRRs, DC increase their ability to present antigen and activate T cells, and to secrete directive cytokines [3], [4]. Simultaneously, they re-organize their asset of integrins, chemokines and chemokine receptors relocating to the draining lymph nodes (LN) to prime T cells [5]. To achieve an optimal organization of the immune response, antigen presenting DC transfer information of the site in which they had encountered the antigenic moiety and of type of pathogen to T cells [6], regulating integrin expression [7], [8].

PRRs, however, are not uniquely expressed by DC or macrophages and TLRs may have a general role in directing cell movement, as supported by the observation that activation of TLR2 and TLR9 regulates CD62L expression in-vitro and modulates trafficking of B cells [9].

By using the CDR3 BV-BJ spectratyping (a PCR-based technique also called “immunoscope”, that allows to identify antigen (ag)-specific, individual T-cell clones within a polyclonal response on the basis of the CDR3 region of the TCR [10]) we observed that trafficking of activated, PLP139-151 (p139)-specific T cells of SJL mice is greatly affected by immunization [11]. Four days after challenge with p139 in IFA enriched with 200 microgrammes/mouse of *M tuberculosis* (*M tb*) (herein, enriched CFA), p139-specific T cells of the pre-immune repertoire (sharing a typical set of TCRs) move to the LN draining the site of injection; at the same time point, T cells of the induced repertoire (characterized by a different TCR repertoire that include a public TCRbeta CDR3 of BV10-BJ1.1 (97b length, herein BV10^+^ cells) [12]) are primed in the draining LN, and 10–14 days after challenge, they reach the spleen, where they replace cells of the pre-immune repertoire.

Also, activated T cells express TLRs; it has been shown that TLR2 acts as co-stimulator during T-cell activation [13], [14], and interferes with T-cell polarization [15], [16] and Treg/Th17 trans-differentiation [17], [18]. However, no data are available on the role of pathogens and PRRs in trafficking of T cells.

As we having shown [11] that re-shuffling of the antigen -specific TCR repertoire depends on the antigen, we hereinafter report that trafficking of p139-specific T cells is regulated by *M tb* in the adjuvant directly through TLR2 expressed on T cells.

## Results

### The Amount of *M tb* in the Adjuvant Modulates Appearance of p139-specific T Cells

**Figure 1:**
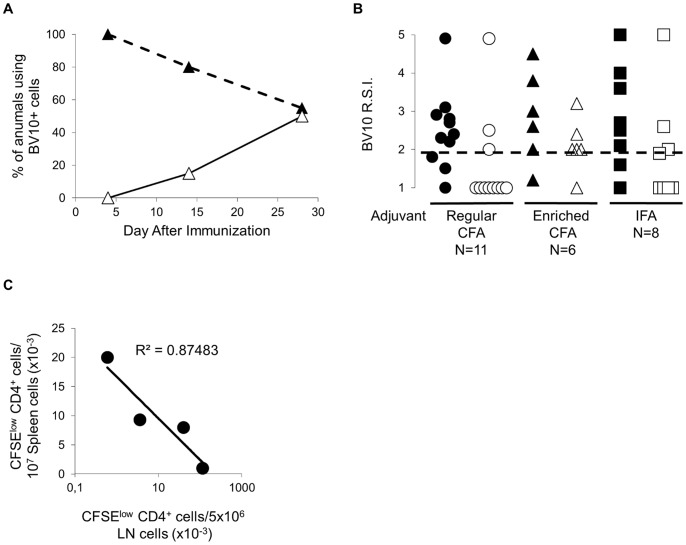
**Amount of M tuberculosis in the adjuvant modulates appearance of p139-specific-T cells in the SJL strain.** SJL mice were immunized with p139 in IFA containing or not 50 or 200 microgrammes/mouse of *M tuberculosis* (regular or enriched CFA, respectively). In all the figures, closed symbols refer to LN cells and open symbols to spleen cells. **A**) Time course of appearance of p139-specific BV10^+^ cells in LN and spleen following challenge with antigen in regular CFA. BV10^+^, p139-specific T cells were measured by immunoscope in draining LN and spleen. **B**) Presence of p139-specific BV10^+^ cells in the spleen at d 14 after s.c. immunization depends on the amount of M tuberculosis in the adjuvant. SJL mice were immunized s.c. with 100 microliters of a 1∶1 suspension of p139 in regular CFA (11 mice), in enriched CFA (6 mice) or in IFA alone (8 mice). Two weeks later, mice were sacrificed and LN and spleen were examined for the presence of p139-specific BV10^+^ cells by immunoscope. Data are reported as R.S.I., and each symbol represents LN or spleen of one mouse, and the dashed line represents the cut off value for positivity in SJL mice. **c**) The number of p139 specific T cells in the spleen 14 d after challenge with peptide in enriched CFA is inversely related to the number of the same cells in the LN. SJL mice were immunized s.c. with p139 in enriched CFA (4 mice). Two weeks later, cells from draining LN and spleen were stained with CFSE and cultured in the presence or absence of 10 microgrammes/ml of p139. After 3 days, cells were recovered and stained with PE-labelled anti CD4 monoclonal antibody. p139-specific cells are calculated as CFSE^low^ CD4^+^ cells in the ag-stimulated sample minus the number of the same cells in the non-stimulated sample.

We immunized SJL mice with p139 in IFA containing 50 microgrammes/mouse of *M tb* (regular CFA), and monitored time of appearance of the shared BV10^+^ cells in draining LN and spleen by CDR3 BV-BJ spectratyping (the so-called “immunoscope”), mirroring the similar experiment performed after immunization of mice with the same amount of peptide but in enriched CFA [11]. Results are shown in Fig. 1A.

All mice showed the presence of BV10^+^ cells in the draining LN by day 4 post-immunization; the same cells were not detected in any spleen at this early time point, similarly to what was observed using enriched CFA as adjuvant [11]. BV10^+^ cells were detected in approximately 90% of draining LN at day 14 post-immunization [12]. Yet, we detected the BV10^+^ cells in the spleen of a minority of the same mice (less than 30%, see also Fig. 1B, p = 0.03), similarly to what we observe in mice challenged with IFA alone (Fig. 1B), and in contrast to what was observed in mice immunized with enriched CFA that consistently showed BV10^+^ cells in the spleen at this time point [11]. This previous result is hereinafter confirmed in Fig. 1B, where 5 out of 6 mice immunized with p139 in the presence of enriched CFA showed BV10^+^ cells in the spleen at day 14 after challenge (p = 1). Fig. 1C shows that an inverse relationship exists between the total number of p139-specific T cells in LN and in the spleen at this time point after immunization in the presence of a high amount of M tb (enriched CFA) in the adjuvant, supporting the idea T cells move from LN to the spleen around day 14 in these latter experimental conditions. Finally, at day 28 post-immunization, BV10^+^ cells were detected in roughly 50% of the spleens of SJL mice immunized with p139, irrespective of the amount of *M tb* in the adjuvant [11].

Thus, appearance of VB10^+^ cells in the spleen of SJL mice immunized s. c. 2 weeks after challenge depends on the administration of high amounts of *M tb* with the antigen.

### Effect of Strain Background and TLR2 Genotype on Sensitivity to Amount of *M tb*

We subsequently examined if the “sensor” for “amount of *M tb*” involved in the modulation of T cell appearance was TLR2, the most important PRRs involved in recognition of *M tb*. A strain lacking this molecule (B6^129-Tlr2tm1kir/3^ (herein B6^tlr2−^) that produces a non-functional, truncated form of TLR2) is available on the B6 background, while the TCR/antigen pair (BV10^+^, p139) we study is restricted to the SJL strain. In a first pilot experiment we examined if F1 (SJLxB6^wt^) mice still use the public BV10^+^ TCR in their response to p139. Two out of 4 F1 (SJLxB6^wt^) mice did so. In order to establish a threshold of significance for antigen-driven expansion observed with the “immunoscope” in the F1 mice, we examined the variability of the peak areas different from the one identifying the shared TCR, in 5 F1 mice. Since the average stimulation index (RSI, see Materials and Methods for a definition) regarding peaks representing TCRs which was not implied in the response to p139 was 1±0.225, we established an RSI ≥1.7 (avg+3 SD) as the significant value for p139-driven expansion of the shared BV10^+^ TCR.

**Figure 2:**
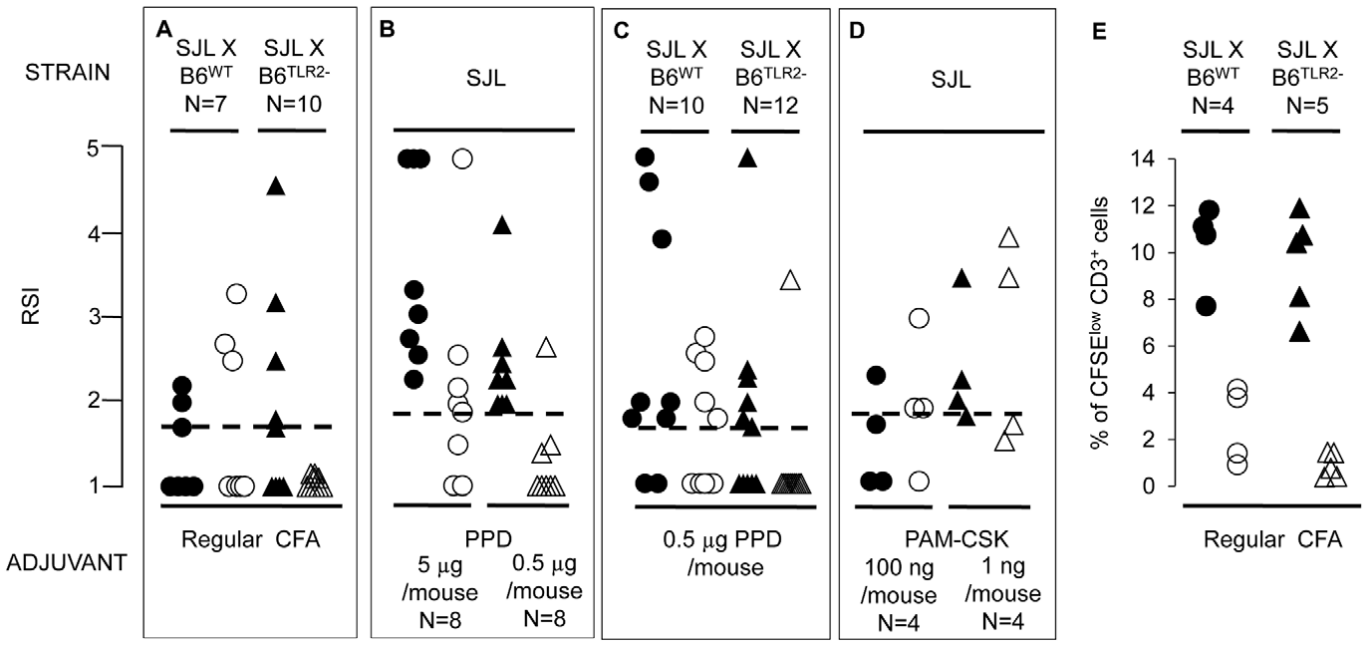
**Mobilization of T cells is strain and TLR2-dependent.** F1 (SJLxB6^wt^), F1 (SJLxB6^tlr−^) (**A**, **C**) or SJL (B, **D**) mice were immunized with p139 in IFA in the presence of the indicated amounts of killed *M tb* (**A**), or of PPD (**B**, **C**) or of a 1∶1 w/w mixture of PAM2-(CSK)3 and PAM3-(CSK)3 (**D**). The number of mice for each group is indicated in the figure. Fourteen days later mice were sacrificed and the presence of T cells carrying the public TCR-beta chain in LN (closed symbols) and spleen (open symbols) was measured by immunoscope. Data are reported as RSI for the peak corresponding to the public BV10 TCR-beta chain for each individual mouse. Dashed lines indicate the cut off value for positivity (established as described in Results). **E**: 4 F1 (SJLxB6^wt^, circles) mice and 5 F1 (SJLxB6^tlr2−^, triangles) mice were immunized with regular CFA in PBS. Two weeks later cells from draining LN (full symbols) and spleen (open symbols) were recovered, labeled with CFSE and cultured in the presence or absence (background) of PPD. After 3 days, the number of CD3^+^ cells that had specifically divided in response to PPD was determined as % of CFSE^low^ CD3^+^ cells over total CD3^+^ cells in the PPD stimulated sample minus % of CFSE^low^ CD3^+^ cells over total CD3^+^ cells in background sample.

Seven F1 (SJLxB6^wt^) mice were immunized s. c. with p139 in regular CFA, in two experiments. Two weeks later, we found that BV10^+^ cells were detected in the spleen at the same frequency of draining LN (3/7, Fig. 2A, left column), although we had used regular CFA as adjuvant.

We then generated F1 (SJL×B6^tlr2−^) mice that displayed a functional TLR2 of SJL origin only. In two independent experiments, 10 F1 (SJL×B6^tlr2−^) mice were immunized with p139 in regular CFA (Fig. 2A, right column) and 5 showed BV10^+^ cells responding to p139 in the LN 14 days post-immunization. In these latter experiments, however, we failed to detect BV10^+^ cells in the spleen of F1 (SJL×B6^tlr2−^) mice (p = 0.03). Thus, the B6 genetic background provides a “highly sensitive”, functionally dominant sensor for *M tb* that confers an “early relocating” phenotype in the presence of a low dose of *M tb* to F1 (SJLxB6^wt^) mice. This “sensor” appears to be TLR2.

PPD provides more selective ligands for TLR2 than the whole *M tb*. We therefore examined if the same observations could be replicated using PPD to supplement IFA. As shown in Fig. 2B, all SJL mice challenged with p139 in the presence of 5 microgrammes of PPD had BV10^+^ cells in LN and 75% of them had BV10^+^ cells also located in the spleen (p = 0.2). When mice were challenged in the presence of 0.5 microgrammes of PPD, BV10^+^ cells were detected in the draining LN of all the mice but in the spleen of only 1 out of 8 mice (p = 0.006). Afterwards, we examined the effect of the TLR2 haplotype on mobilization of BV10^+^ cells upon challenge in the presence of PPD (Fig. 2C). Seven out of 10 F1 (SJL×B6^wt^) mice challenged with p139 in the presence of 0.5 microgrammes of PPD showed an expansion of BV10^+^ cells in the draining LN, and 5 also showed expansion of these cells in the spleen (p = 0.6). When 12 F1 (SJL×B6^tlr2−^) mice were immunized in this experiment, 6 showed the expansion of BV10^+^ cells in draining LN, but only 1 showed it in the spleen (p = 0.07). Thus, we substantially reproduced the modulation of T-cell mobilization due to whole *M tb* using PPD alone as a supplement, and we observed the same genetic effect on appearance of effector T cells.

To formally prove that engagement of TLR2 is sufficient to drive mobilization of T cells, we supplemented IFA with a mixture of PAM2-(CSK)3 and PAM3-(CSK)3, (selective ligands for TLR2/1 and TLR2/6 heterodimers, respectively). As shown in Fig. 2D, the addition of PAM-(CSK)3 to IFA is sufficient to mobilize p139-specific T cells in 7/8 mice, tested at 100 or 1 ng/mouse, as compared to administration of p139 in IFA alone, shown in Fig. 1B.

An important question is whether the dependence of T-cell mobilization on TLR2 haplotype is limited to this model antigen (or even only to T-cells carrying the studied CDR3-beta chain), or rather this mechanism has a general relevance and applies to any antigen. To address this point, in a separate experiment we immunized 4 F1 (SJLxB6^wt^) mice and 5 F1 (SJLxB6^tlr2−^) mice with regular CFA. Two weeks later cells from draining LN and spleen were recovered, labeled with CFSE and cultured in the presence or absence (background) of PPD. After 3 days, the fraction of CD3^+^ cells that had specifically divided in response to PPD was determined as % of CFSE^low^ CD3^+^ cells over total CD3^+^ cells. Results, reported in Fig. 2E, showed that the two groups of F1 mice had the same number of PPD-specific T-cells in the LN. T cells specific for PPD were present in consistent amount in 2 out of 4 spleens obtained from F1 (SJLxB6^wt^) mice, while spleens from all 5 F1 (SJLxB6^tlr2−^) mice displayed very low numbers of T-cells proliferating in response to PPD. Thus, it appears that TLR2 haplotype regulates the appearance in the spleen also of activated T cells specific for allo-antigen(s) of bacterial origin, and the observations reported here are not limited to self-reactive immune responses.

It may be suggested that challenge in enriched CFA may promote leakage of peptide and adjuvant from draining LN, and that emergence of p139-specific T cells in the spleen, in this case, may be the result of independent priming. However, results previously reported [11] and those herein shown (time progression in identification of T cells sharing the same TCR in LN first and then in the spleen, an inverse relationship between p139-specific T cells in draining LN and in the spleen, and dependence of appearance on TLR2 haplotype) considered together, support the hypothesis that the same T cells, defined by their TCR usage, when activated in the LN, relocate to the spleen with different kinetic depending on amount of *M tb* in the adjuvant and TLR2 haplotype.

### A single Non-Synonymous Polymorphism of TLR2 is Responsible for Early/late Appearance of T Cells

In order to examine the differences of TLR2 in B6 and SJL strains reflected by mobilization of the T cells, we sequenced TLR2 of SJL and determined its level of expression on activated T cells and APC.

**Figure 3:**
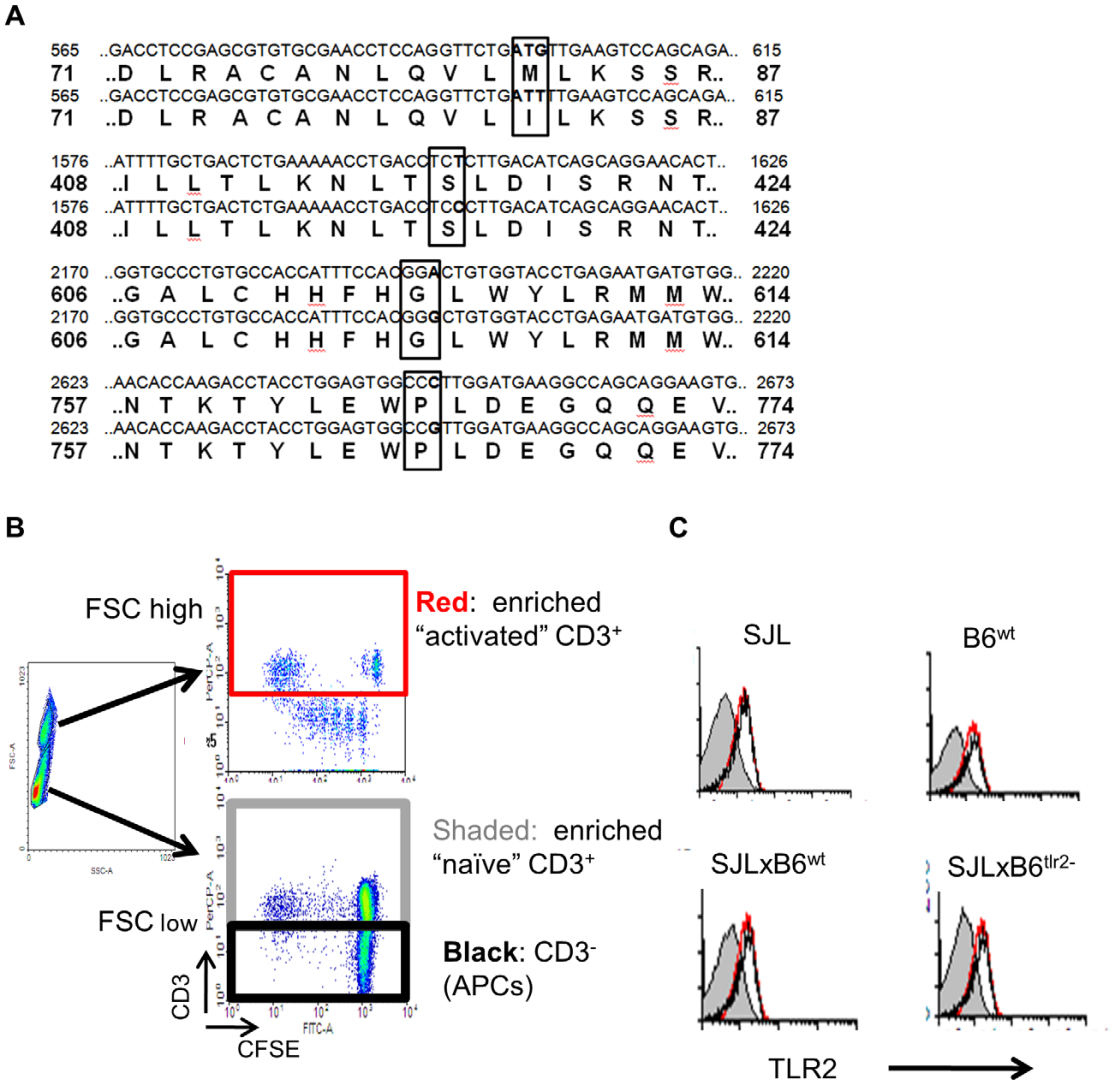
**Identification of polymorphisms of TLR2 between SJL and B6 strains and expression of TLR2 on immune cells.** **A**) Comparison of the sequence of TLR2 of SJL and B6. The non-synonymous and synonymous polymorphisms are boxed. Base (first line) and aminoacid (second line) sequences of B6 and the corresponding sequences (third and fourth lines, respectively) of SJL around the polymorphic residues are reported. **B**) Identification of enriched activated T cells, naïve T cells and APC in LN cells by scattering properties. SJL, B6^wt^, F1 (SJLxB6^wt^) and F1 (SJLxB6^tlr2−^) mice were immunized with IFA containing 50 microgrammes/mouse of *M tb*.. 8 days later, mice were sacrificed and cells from draining LN were loaded with CFSE and stimulated with PPD. After 3 days, cells were collected and stained with PE-labeled anti-CD3 monoclonal antibody. The colours define cells examined for TLR2 expression in panel C. **C**) SJL, B6^wt^, F1 (SJLxB6^wt^) and F1 (SJLxB6^tlr2−^) mice were immunized with IFA containing 50 microgrammes/mouse of *M tb*. Eight d later, mice were sacrificed and cells from draining LN were stimulated with PPD. After 3 days, cells were collected and stained with PE-labeled anti-CD3 mAb and with FITC labeled anti-TLR2 mAb. Expression of TLR2 was evaluated on high scattering CD3^+^ cells (activated T cells, red line), low scattering CD3^+^ cells (naïve T cells, shaded area) and CD3^−^ cells (mostly APC, black line), as shown in **B**.

We sequenced the cDNA encoding for TLR2 from mice of SJL and B6 strains bred in our facility, as described in the Materials and Methods. The TLR2 sequence of B6 mice used in this study is the same as the one deposited at the NCBI (http://www.ncbi.nlm.nih.gov, accession numbers AK005043.1 for mRNA and BAB23770.1 for a sequence). The sequence obtained from the SJL mice displays 3 synonymous polymorphisms and one non-synonymous polymorphism, 83Met (B6) to Ile (SJL), in the second LRR (Fig. 3A).

We next evaluated if the TLR2 polymorphism determined a modification of its level on the cell surface of APC or of activated T cells. We immunized SJL, B6^wt^, F1 (SJL×B6^wt^) and F1 (SJL×B6^tlr2−^) with CFA, collected cells from draining LN 4 days after immunization, when specific T cells still appear to be restricted to draining LN ( [11] and Fig. 1A). Cells were stimulated with PPD and stained for CD3 and TLR2. Results are shown in Fig. 3B and C. According to forward scattering and CD3 expression, LN cells were divided in APC, activated T cells and naïve T cells (Fig. 3B). The level of expression of TLR2 (Fig. 3C) was the same on APC and activated T cells in SJL, B6 and (SJL×B6^wt^) mice. A slightly lower expression (a difference of ≈ 20% in the median fluorescence channel) was found on the surface of T cells and APC obtained from F1 of SJL×B6^tlr2−^.

**Figure 4:**
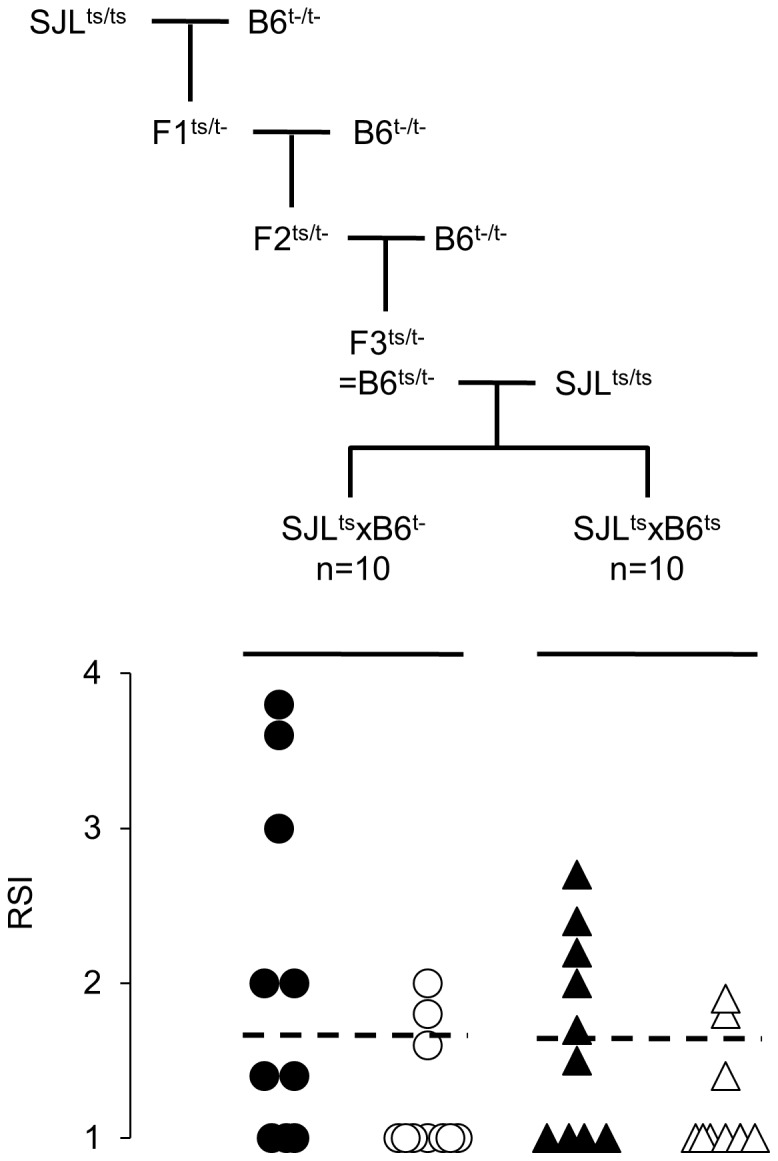
**Mobilization of T cells depends on the polymorphism of TLR2.** Upper section: transfer of TLR2 of SJL onto B6 background. SJL mice (homozygous for TLR2 of SJL, ts+/+) mice were crossed with B6^tlr2−^ (t-). F1 mice (genotype ts/t-) were then backcrossed with B6^tlr2−^ and F2 mice of genotype ts/t- are further backcrossed with B6^tlr2−^ generating F3 mice ts/t- that share ≈90% of their genome with B6^wt^. These latter mice (B6^ts/t−^) were crossed with SJL mice to generate TLR2-heterozygous F1 (SJL^ts^xB6^t−^) and TLR2-homozygous F1 (SJL^ts^xB6^ts^) mice. Lower section: these two groups of littermates were immunized with p139 in regular CFA and 14 days later the presence of T cells carrying the public TCR-beta chain in LN (closed symbols) and spleen (open symbols) was examined by immunoscope, as described above. Ten mice per group were examined, and mice of each group derived from two distinct (B6^ts/t−^×SJL) couples. Data are reported as RSI for the peak corresponding to the public TCR-beta chain for each individual mouse. The cut off value for the RSI at 1.7 is indicated.

In order to formally establish if the difference in sequence of TLR2 regulates the mobilization of T cells in response to regular CFA, we produced F1 mice having two copies of the TLR2 gene of SJL origin (F1 (SJL^ts^×B6^ts^)). The procedure is shown in Fig. 4. “B6^ts/t−^” mice (i.e. mice of B6 background heterozygous for TLR2 of SJL (ts) and B6^tlr2−^ (t-) origin) were bred with SJL mice, producing two types of F1 littermates, (SJL×B6^tlr2−^) and (SJL×B6^ts^), the former having one copy and the latter two copies of TLR2 of SJL origin. Results show that F1 mice (differing in gene load of TLR2 of SJL origin) display the same phenotype, and only 20% of the mice display a presence of these cells in the spleen in both groups, in contrast to 50% of the mice showing it in the LN. Thus, residue at position 83 (Met to Ile) of TLR2 is critical to determine “late” versus “early” appearance phenotype, while its gene load does not have a role.

### T-cell Mobilization is Regulated by TLR2 Expressed on T Cells

We next sought to understand if T-cell mobilization is regulated by TLR2 expressed on APCs or directly by TLR2 expressed on T cells.

**Figure 5:**
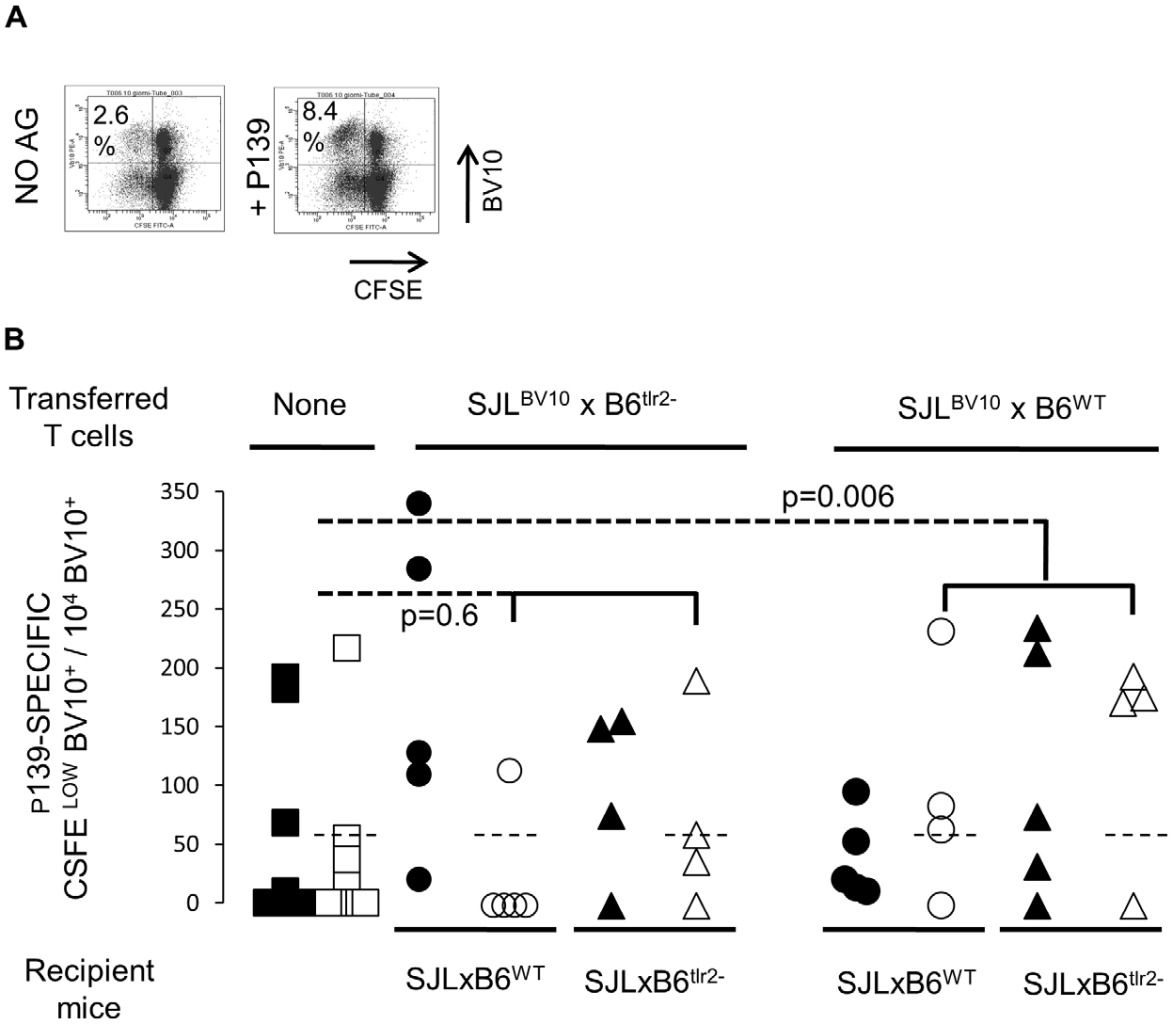
**Appearance of T cells in the spleen is regulated by TLR2 expressed on T cells.** **A**Detection of p139-specific T cells in SJL^BV10^ mice. One SJL^BV10^ mouse (at the 6^th^ backcross onto SJL background) was immunized s. c. with p139 in regular CFA, as described above. Ten days later LN cells were obtained, stained with CFSE and cultured in the presence or absence of p139. The number of BV10^+^, p139-specific T cells in the draining LN is measured as the number of CFSE^low^ BV10^+^ cells in the ag-stimulated sample, minus the number of the same cells in the non-stimulated sample. **B**) T cells were enriched from the spleen of naïve F1 mice of (SJL^BV10^×B6^wt^) or (SJL^BV10^×B6^tlr2−^), and transferred i. p. into naïve F1 mice of (SJL×B6^wt^) or (SJL×B6^tlr2−^). A week later, recipient mice were immunized s. c. with p139 in regular CFA, and after, further 14 days, cells from LN and spleen were prepared, and the presence of BV10^+^, p139-specific T cells was measured as described in Fig. 4a. The 11 non-transferred mice were 6 F1 (SJL×B6^wt^) mice and 5 F1 (SJL×B6^tlr2−^) mice, all challenged with p139 in regular CFA. Data are expressed as the number of BV10^+^, CFSE^low^, p139-specific cells/10^4^ BV10^+^ cells.

In order to trace p139-specific T cells, we prepared a transgenic mouse expressing the shared beta chain BV10-CASS SGS NTEV-BJ1.1 on the SJL background (SJL^BV10^). To detect T cells expressing the transgenic beta chain with available anti-BV10 monoclonal antibodies, we used the BV10 chain of the TCR type 2 haplotype as a backbone. Transgenic T cells proliferating in response to p139 after immunization can be detected by flow cytometry as BV10^+^ CFSE^low^ cells (Fig. 5A).

We crossed SJL^BV10^ mice with B6^wt^ or with B6^tlr2−^. T cells from these F1 mice were then transferred into naïve F1 (SJLxB6^wt^) and F1 (SJL×B6^tlr2−^), and recipient mice were challenged with p139 in regular CFA a week later. LN and spleen cells were collected after14 days, stimulated with peptide and cells, proliferating in response to p139, were measured as BV10^+^ CFSE^low^ cells as described in Materials and Methods.

In these experiments, transferred BV10^+^ T cells possess functional TLR2 only of the SJL genotype (donors F1 SJL^BV10^×B6^tlr2−^), or of both strains (donors F1 SJL^BV10^×B6^wt^); the asset of TLR2 of APC and stromal cells is dictated by the TLR2 haplotype of receiving mice. Results are reported in Fig. 5B.

Since the TCR locus of B6 mice also belongs to the type2 haplotype, we first established the background reading of spleen of both F1 recipient mice immunized with p139. The number of BV10^+^ CFSE^low^ cells in the spleen after stimulation with p139 was ≤55/10^4^ BV10^+^ cells in 10 out of 11 mice (Fig. 5B), and we used this as a threshold value.

When we transferred T cells obtained from F1 (SJL^BV10^×B6^tlr2−^) mice (having only TLR2 of SJL origin), the number of BV10^+^ cells in the spleen of recipient mice was similar to that observed in control non-transferred mice, irrespective of the TLR2 haplotype of recipient mice (p = 0.6). On the other hand, when transferred T cells had been obtained from F1 (SJL^BV10^×B6^wt^) mice that had TLR2 of both SJL and B6 origin, 75% of the spleens of recipient mice showed a number of BV10^+^ CFSE^low^ cells significantly higher than in control mice (p = 0.006), once again irrespective of the TLR2 haplotype of receiving mice.

Thus, appearance of T cells in the spleen 14 days after s. c. immunization was modulated by TLR2 of transferred T cells, while TLR2 expressed on APCs and stromal cells did not appear to play a role.

## Discussion

We herein report that the time by which antigen-specific T cells appear in the spleen following priming in LN is modulated by concentration of *M tb* in the adjuvant directly on activated T cells. The modulation is strain-dependent and one non-synonymous polymorphism between TLR2 expressed on T cells of SJL and B6 accounts for “late” versus “early” appearance phenotype.

An appropriate adaptive T-cell mediated immune response is necessary to obtain clearance of the pathogen, preventing a chronic evolution of the infection (e.g. [19]). In the present work, we studied the appearance of T cells in the spleen following priming in the LN, and used it as a marker of mobilization of T cells. Further studies are required to assess if TLR2 directly influences also trafficking of T cells to other sites, or if a passage through the spleen is necessary to licence T cells to further diffuse to peripheral organs. The microenvironment of the spleen has distinct properties with respect to that of LN [20], and trafficking through the spleen seems necessary for myelin-specific T cells to acquire encephalitogenic properties [21]. We have observed that T cells display some differences in homing to the CNS upon encephalitogenic challenge that depend on the TLR2 haplotype (manuscript in preparation). However, invasion of the CNS and the following EAE depend upon the administration of a high dose of CFA, and these data were obtained in experimental conditions different from those used in this work. Therefore, at present we cannot establish a direct link between the observations reported here about the role of *M tb* and TLR2 in modulating relocation of p139-specific T cells from LN to spleen, and T-cell trafficking into the CNS.

It has recently been reported that Central Nervous System-specific T-cell blasts need to traffic through the lung in order to acquire migratory properties that allow them to home to the spleen first and then to the CNS [22]. Our observations reported here suggest that components of bacterial origin, administered in our experimental model with the adjuvant, are needed for T cells to be licensed to move to the spleen. In the case of T cells activated in vitro, the passage through the lungs may provide the possibility to encounter bacteria able to engage TLR2 and thereby acquire the appropriate migratory phenotype. Endogenous ligands of TLR2 [23] may also be involved in the modulation of trafficking properties of activated T cells, and regulation of these endogenous ligands may promote or protect from CNS invasion.

TLRs are key molecules for the determination of the Th phenotype. Activation of DC via TLRs plays a major role in the polarization of naïve T cells, and direct engagement of TLR2 on activated T cells modulates the trans-differentiation between FoxP3^+^ and Th17 cells [17], allowing the immune response to adapt to the presence of infectious agents without maintaining constitutively a pro-inflammatory aptitude.

Modulation of the trafficking properties of immune cells is an effect of PRRs engagement, with the paramount example of the well-studied cycle of DC [5]. Activation of TLR2 and TLR9 regulates CD62L expression in B cells modifying the distribution of B cells between spleen and LN [9]. Here, we report that TLR2 also regulates the time by which antigen-specific T cells mobilize in vivo, showing that pathogen-derived motives can modulate the trafficking properties of T cells without mediation by APCs.

A trivial explanation of our observation would be that TLR2 polymorphism and *M tb* in the adjuvant influence the down-regulation of CD62L (as it occurs in B cells [9]) and, consequently, the relocation of activated T cells to the spleen. However, this is not the case; In fact, while the haplotype of TLR2 has some effect on the down-modulation of CD62L on activated T cells, the amount of *M tb* in the adjuvant does not regulate the down-modulation of CD62L in the SJL strain (not shown). We also observed that the level of expression of Sphingosin 1P-R1 (a relevant molecule involved in egress of T lymphocytes from the LN [24]) does not vary depending on the TLR2 polymorphism. Thus, neither of these 2 molecules appears to represent the common mechanism underlying the observations herein reported.

T-cell activation is accompanied by a modification of the cell surface asset of many molecules involved in the regulation of cell trafficking. Several of these molecules are differentially expressed on naïve, effector and memory-T cells, and regulate the trafficking properties of each T-cell group. Further studies will be needed to establish which, among the molecules regulating T-cell trafficking, are modulated by TLR2 to lead to the differences in T-cell mobilization reported here.

A large array of PRRs is expressed in T-cells, and the integration of signals coming from each PRR determines the final effect of encounters with microorganisms able to interact with several of them. Molecules belonging to the family of NOD-like receptors play an important role in the recognition of *Mtb*. Analogous to TLRs, it has been reported that molecules belonging to this family are also expressed in T cells (see, e.g., [25], [26]), with conflicting evidences about their role [27], [28]. Nod1 and Nod2 interplay with TLRs in macrophages [29], and, more relevant to the present model, Nod1 cooperates with TLR2 to promote the activation of CD8^+^ cells [30]. It will be of interest to assess if molecules belonging to this family co-operate with TLR2 also to modulate T cell mobility.

The time by which effector-T cells reach the site of infection is crucial for an effective pathogen control. Polymorphisms of TLRs have been associated to a susceptibility or resistance to infectious diseases (e.g. [2]). In several cases, a link between polymorphism and Th polarization has been demonstrated and suggested to sustain differences in pathogen control. TLR2 polymorphism 83Met/Ile alters the promptness of mobilization of T cells upon limiting concentration of the ligand, suggesting another mechanism for polymorphisms of PRRs to affect the control of infections.

Mobilization of self-reactive T cells from survival niches also plays an important role in the pathogenesis of autoimmune diseases, in particular of those in which a poorly predictable degree of relapses strongly influences the final outcome of the disease. It is a common observation that the occurrence of infections often leads to flares of autoimmune diseases, and a prevention of the release of T cells from LN is now an effective therapy [24]. Bacteria colonising mucosal surfaces [31] also provide an essential contribution to the pathogenesis of autoimmune disorders by regulating the polarization of T helper cells. Our observation adds a further mechanism to the role of infections in autoimmunity, suggesting that products derived from bacteria may contribute to mobilization of activated self-reactive T cells from niches that allow them to escape natural (e.g., the spontaneous remission during Multiple Sclerosis) or therapy-induced (as in Rheumatoid Arthritis [32]) control.

Several environment-derived and endogenous stimuli cooperate during activation of T cells by engaging a variety of PRRs, and thereby leading to effective control of infectious agents, or to their chronic containment, or in some cases to self-reactivity. The ability of pathogens engaging TLR2 directly on activated T cells through a polymorphic site, to regulate trafficking of CNS-specific, effector T cell in vivo has been the subject of our study.

## Materials and Methods

### Mice, Peptide and Immunization

Breeding couples for SJL, C57Bl/6 (B6^wt^) mice and B6^129-Tlr2tm1kir/3^ (B6^tlr2−^) were purchased from Charles River, Calco, Italy, and bred in the animal housing facility of the Universita’ Cattolica del S. Cuore in Rome. B6 mice, heterozygous for TLR2 of SJL (B6^ts/t−^), were generated as described in Fig. 4, by back-crossing F1 (SJLxB6^tlr2−^) onto B6^tlr2−^ for 2 generations.

PLP139-151 (Ser 140 [33]) was purchased from PRIMM (Milan, Italy) and was >95% pure, as determined by HPLC and mass-spectroscopy.

Mice were immunized subcutaneously (s. c.) with 50 microgrammes/mouse of p139 in PBS, emulsified 1∶1 with Incomplete Freund’s Adjuvant (IFA), complete (CFA) Freund’s adjuvant (CFA is IFA containing 1 mg/ml of killed and heat dried *M. tb* H37RA, i.e. each mouse received 50 microgrammes of *M. tb* H37RA) or IFA containing 4 mg/ml of killed and heat dried *M. tb* H37RA (enriched CFA, i.e. each mouse received 200 microgrammes of *M. tb* H37RA) (Sigma-Aldrich, St Louis, MO, USA) in a final volume of 100 microliters/mouse, or in the conditions indicated in Figure 2. All experimental procedures involving animals were approved by the internal Ethics Committee.

### TLR2 Sequencing

cDNA was prepared from LN cells of SJL and B6 mice and sequenced from PCR products using 5 pairs of primers covering its sequence from b376 (aa 8) to b2706 (aa 784), using a Genetic Analyzer 3130 (Life Technologies, The Netherlands) and analyzed with Chromas Lite 2.1 free software. To sequence TLR2, cDNA obtained from spleen cells was submitted to RT-PCR (35 cycles) using five couple of primers (see below). PCR products were purified using a gel extraction kit (Qiagen), and conjugated with the BigDYE 3.1 (Life Technologies, The Netherlands), in compliance with manufacturer’s instructions.

### Primer Pairs

TLR2 FW1 366 tctttggctcttctggatcttggtg

TLR2 REV1aaaaatctccagcaggaaagcagac1005

TLR2 FW2886gctgggctgacttctctcaatg

TLR2 REV2acagtcgtcgaactctacctcc 1214

TLR2 FW31199 agagttcgacgactgtaccctc

TLR2 REV3gaatgcacgtttttaccacccg1720

TLR2 FW41623acttttcatccgatgcccgac

TLR2 REV4acccaatgggaatcctgctcac 2318

TLR2 FW52271 tgctatgatgcctttgtttcc

TLR2 REV5ttgcagttctcagatttaccc 2698

Numbers refer to the deposited sequence (http://www.ncbi.nlm.nih.gov, accession numbers AK005043.1).

### T-cell Receptor Repertoire Analysis

Repertoire analysis was performed as previously described [12], [34], [35]. Briefly, 5×10^6^ LN cells or 10^7^ spleen derived cells/well were cultured in the presence or absence of 10 microgrammes/ml of p139 for three days in RPMI-1640 medium (Sigma- Aldrich, St Louis, MO, USA) supplemented with 2mM L-glutamine, 50 microM 2-ME, 50 microgrammes/ml gentamicin (Sigma- Aldrich, St Louis, MO, USA), and 1% mouse serum. Three days later, cells were collected, washed in PBS and resuspended in RLT. Total RNA was isolated from cell suspensions using RNeasy Mini Kit (Qiagen GmbH, Hilden, Germany) according to the manufacturer’s instructions. cDNA was synthesized using an oligo-dT primer (dT15) (Gibco BRL Life Technologies, Basel, Switzerland). cDNA was subjected to PCR amplification using a common Constant (C) beta primer (CACTGATGTTCTGTGTGACA) in combination with the variable beta (BV) primers previously described [12]. Using 2 microliters of this product as a template, run-off reactions were performed with a single internal fluorescent primer for each BJ tested (previously described [12]). These products were then denatured in formamide and analyzed on an Applied Biosystem 3130 Prism using Gene-mapper v4.0 software (Applied Biosystem, Foster City, CA, USA). Results are also reported as R.S.I. (rate stimulation index = normalized peak area obtained from cells stimulated with Ag/normalized peak area of non-stimulated cells).

### TLR2 Staining

Surface expression of TLR2 was determined by FACScan. SJL, B6^wt^, F1 (SJL×B6^wt^) and F1 (SJL×B6^tlr2−^) mice were immunized with regular CFA. Four days later, LN cells were obtained, stained or not with CFSE and stimulated with PPD for 3 days. Cells were then divided in aliquots and labelled with the Rat antimouse AntiCD282 (AbD Serotec Raileigh NC (USA)) mAb and PE-labelled anti CD3 mAb (BD Bioscience Pharmingen, San Diego, CA, USA) and read on a BD FACScan flow cytometer.

### Preparation of BV10 Transgenic Mice

In our previous work [12] we identified a CDR3-beta chain (BV10-BJ1.1 of sequence CASS SGS NTE) consistently expressed on T cells specific for p139 in the SJL mouse. In order to have a reliable source of p139-specific T cells carrying this rearrangement, we prepared a transgenic mouse carrying a gene encoding for its TCR-beta chain using the BV10 sequence of the TCR2 haplotype as a backbone, to identify cells expressing the transgene by available anti-BV10 antibodies and vector pP142b8AR (a kind gift from H. Pircher [36]).

Founder transgenic mice were prepared at PolyGene AG (Rumlang, CH). Briefly, the gene segment encoding for BV10-CASS SGS NTE-BJ1.1 was inserted into the pP142b8AR vector. The resulting TCR-beta chain was cloned into a plasmid harboring this T-cell receptor beta chain (TCRβ) cDNA driven by the H-2K promoter/immunoglobulin heavy chain enhancer (pHSE3’-tgbeta). A 6.3 kbp DNA fragment from pHSE3’-tgbeta was excised with the restriction endonuclease XhoI. The fragment was purified from SeaKem GTG agarose (avoiding exposure to UV light) using the Qbiogene Geneclean Spin kit, dialysed 24 h against 2 l microinjection buffer (10 mM Tris.HCl pH 7.2, 0.1 mM EDTA), and finally diluted to a concentration of 1.1 ng/µl. In four distinct rounds, the DNA was injected into B6N-derived zygotes. For this purpose, B6N female mice were obtained from Charles River WIGA Sulzfeld, superovulated at 45 to 47 days of age and mated in the PolyGene mouse facility to B6N breeder males, originally also obtained from Charles River.

Injected zygotes were cultivated overnight and transferred into pseudopregnant B6×CBA F1 females, also from Charles River. Animals were kept in individually ventilated cages.

Tail DNAs of 3 out of 46 littermates were found positive for the transgenic TCR-beta chain. The 3 mice were mated with the B6N breeder and a transgene-positive pup was selected as founder of the colony.

The founder was then transferred to the housing facility of the Catholic university and mated with B6 mice to establish a founder colony of transgenic mice on the B6 background. Once the colony was established, we back-crossed the transgenic TCR BV10 chain from B6 to SJL mice. All the experiments reported in the present work and involving VB10-transgenic mice were performed using mice that had been back-crossed for at least 6 generations into the SJL background (herein SJL^VB10^), in most cases 9 generations or more.

Transgenic mice were bred on a RAG1/RAG2^wt^ background to allow free rearrangement of the alpha-chain. Naïve heterozygous SJL^BV10^ mice express the transgenic beta chain on 62–68% of both CD4^+^ and CD8^+^ cells. Confirming results obtained in wild type SJL (SJL^wt^), transgenic BV10^+^ T cells do not participate in the response to p139 that arises spontaneously in the SJL mouse [11] but are activated only following immunization with peptide antigens. BV10^+^ T cells that divided in response to in-vitro stimulation with p139 are 15% and 23% of total BV10^+^ cells at day 4 (not shown) and 10 after immunization, respectively (see also Fig. 4A of the main text). Frequency of p139-specific precursors could be estimated to be between 0.5 and 0.15% of BV10^+^ cells in the naïve SJL^BV10^ mouse.

In order to identify p139-specific VB10 transgenic cells we followed the protocol described in [11]. Cells from SJL^VB10^ mice were stained with CFSE (Invitrogen, Eugene, OR, USA) and stimulated in vitro with p139. Three days later cells were stained with a PE-labelled anti mouse BV10 mAb (BD Bioscience Pharmingen, San Diego, CA, USA). T cells expressing the BV10 transgenic chain and recognizing p139 were detected as BV10^+^ CFSE^low^ cells (Fig. 5A) on a BD FACScan flow cytometer.

### Transfer of Naïve T Cells

SJL^VB10^ mice were mated with B6^wt^ or B6^tlr2−^ and spleen cells were prepared from 7–10 week old naïve F1 mice. T cells were enriched by depleting spleen adherent cells and B220^+^ cells, using anti-B220 MACS selection Kit (Milteny Biotech GmBH, Bergish Gladbach, Germany). The final population was composed of 83% CD4^+^ or CD8^+^ cells and the fraction of BV10^+^ cells was 49.8%. Five ×10^6^ enriched T cells were transferred i. p. into naïve mice. One week later recipient mice were immunized as described above and sacrificed 14 d later. Cells from draining LN and spleen were stained with CFSE, cultured for 3 days in the presence (test) or absence (control) of p139 and stained with PE-labelled anti BV10 mAb. The number of transgenic T cells specific for p139 was calculated as the number of BV10^+^ CFSE^low^ cells in the sample stimulated with p139 minus the number of the BV10^+^ CFSE^low^ cells in the control sample, both values normalized to 10^4^ total VB10^+^ cells.

### Statistical Analysis

Data were compared using two-tailed Fisher exact test.

## References

[1] Ley K, Morris M (2005) Signals for lymphocyte egress. Nature immunology 6: 1215–1216. 16369564 10.1038/ni1205-1215

[2] Ma X, Liu Y, Gowen BB, Graviss EA, Clark AG, et al (2007) Full-exon resequencing reveals toll-like receptor variants contribute to human susceptibility to tuberculosis disease. PLoS One 2: e1318. 18091991 10.1371/journal.pone.0001318 PMC2117342

[3] Guery JC, Ria F, Galbiati F, Smiroldo S, Adorini L (1997) The mode of protein antigen administration determines preferential presentation of peptide-class II complexes by lymph node dendritic or B cells. International immunology 9: 9–15. 9043943 10.1093/intimm/9.1.9

[4] Ria F, Penna G, Adorini L (1998) Th1 cells induce and Th2 inhibit antigen-dependent IL-12 secretion by dendritic cells. European journal of immunology 28: 2003–2016. 9645382 10.1002/(SICI)1521-4141(199806)28:06<2003::AID-IMMU2003>3.0.CO;2-S

[5] Macagno A, Napolitani G, Lanzavecchia A, Sallusto F (2007) Duration, combination and timing: the signal integration model of dendritic cell activation. Trends in immunology 28: 227–233. 17403614 10.1016/j.it.2007.03.008

[6] Mora JR, von Andrian UH (2006) T-cell homing specificity and plasticity: new concepts and future challenges. Trends in immunology 27: 235–243. 16580261 10.1016/j.it.2006.03.007

[7] Dudda JC, Simon JC, Martin S (2004) Dendritic cell immunization route determines CD8+ T cell trafficking to inflamed skin: role for tissue microenvironment and dendritic cells in establishment of T cell-homing subsets. Journal of immunology 172: 857–863. 10.4049/jimmunol.172.2.857 14707056

[8] Stagg AJ, Kamm MA, Knight SC (2002) Intestinal dendritic cells increase T cell expression of alpha4beta7 integrin. European journal of immunology 32: 1445–1454. 11981833 10.1002/1521-4141(200205)32:5<1445::AID-IMMU1445>3.0.CO;2-E

[9] Morrison VL, Barr TA, Brown S, Gray D (2010) TLR-mediated loss of CD62L focuses B cell traffic to the spleen during Salmonella typhimurium infection. Journal of immunology 185: 2737–2746. 10.4049/jimmunol.1000758 PMC3745606 20660707

[10] Ria F, van den Elzen P, Madakamutil LT, Miller JE, Maverakis E, et al (2001) Molecular characterization of the T cell repertoire using immunoscope analysis and its possible implementation in clinical practice. Current molecular medicine 1: 297–304. 11899078 10.2174/1566524013363690

[11] Penitente R, Nicolo C, Van den Elzen P, Di Sante G, Agrati C, et al (2008) Administration of PLP139–151 primes T cells distinct from those spontaneously responsive in vitro to this antigen. J Immunol 180: 6611–6622. 18453580 10.4049/jimmunol.180.10.6611

[12] Nicolo C, Di Sante G, Orsini M, Rolla S, Columba-Cabezas S, et al (2006) Mycobacterium tuberculosis in the adjuvant modulates the balance of Th immune response to self-antigen of the CNS without influencing a “core” repertoire of specific T cells. International immunology 18: 363–374. 16415105 10.1093/intimm/dxh376

[13] Cottalorda A, Verschelde C, Marcais A, Tomkowiak M, Musette P, et al (2006) TLR2 engagement on CD8 T cells lowers the threshold for optimal antigen-induced T cell activation. European journal of immunology 36: 1684–1693. 16761317 10.1002/eji.200636181

[14] Komai-Koma M, Jones L, Ogg GS, Xu D, Liew FY (2004) TLR2 is expressed on activated T cells as a costimulatory receptor. Proceedings of the National Academy of Sciences of the United States of America 101: 3029–3034. 14981245 10.1073/pnas.0400171101 PMC365739

[15] Liu H, Komai-Koma M, Xu D, Liew FY (2006) Toll-like receptor 2 signaling modulates the functions of CD4+ CD25+ regulatory T cells. Proceedings of the National Academy of Sciences of the United States of America 103: 7048–7053. 16632602 10.1073/pnas.0601554103 PMC1444884

[16] Sutmuller RP, den Brok MH, Kramer M, Bennink EJ, Toonen LW, et al (2006) Toll-like receptor 2 controls expansion and function of regulatory T cells. The Journal of clinical investigation 116: 485–494. 16424940 10.1172/JCI25439 PMC1332026

[17] Nyirenda MH, Sanvito L, Darlington PJ, O’Brien K, Zhang GX, et al (2011) TLR2 stimulation drives human naive and effector regulatory T cells into a Th17-like phenotype with reduced suppressive function. Journal of immunology 187: 2278–2290. 10.4049/jimmunol.1003715 21775683

[18] Reynolds JM, Pappu BP, Peng J, Martinez GJ, Zhang Y, et al (2010) Toll-like receptor 2 signaling in CD4(+) T lymphocytes promotes T helper 17 responses and regulates the pathogenesis of autoimmune disease. Immunity 32: 692–702. 20434372 10.1016/j.immuni.2010.04.010 PMC2878917

[19] Holley MM, Kielian T (2012) Th1 and Th17 cells regulate innate immune responses and bacterial clearance during central nervous system infection. Journal of immunology 188: 1360–1370. 10.4049/jimmunol.1101660 PMC3709259 22190181

[20] Wrenshall L (2003) Role of the microenvironment in immune responses to transplantation. Springer seminars in immunopathology 25: 199–213. 12955467 10.1007/s00281-003-0138-y

[21] Flugel A, Berkowicz T, Ritter T, Labeur M, Jenne DE, et al (2001) Migratory activity and functional changes of green fluorescent effector cells before and during experimental autoimmune encephalomyelitis. Immunity 14: 547–560. 11371357 10.1016/s1074-7613(01)00143-1

[22] Odoardi F, Sie C, Streyl K, Ulaganathan VK, Schlager C, et al (2012) T cells become licensed in the lung to enter the central nervous system. Nature 488: 675–679. 22914092 10.1038/nature11337

[23] Erridge C (2010) Endogenous ligands of TLR2 and TLR4: agonists or assistants? Journal of leukocyte biology 87: 989–999. 20179153 10.1189/jlb.1209775

[24] Cohen JA, Chun J (2011) Mechanisms of fingolimod’s efficacy and adverse effects in multiple sclerosis. Annals of neurology 69: 759–777. 21520239 10.1002/ana.22426

[25] Lech M, Avila-Ferrufino A, Skuginna V, Susanti HE, Anders HJ (2010) Quantitative expression of RIG-like helicase, NOD-like receptor and inflammasome-related mRNAs in humans and mice. International immunology 22: 717–728. 20584763 10.1093/intimm/dxq058

[26] Petterson T, Mansson A, Riesbeck K, Cardell LO (2011) Nucleotide-binding and oligomerization domain-like receptors and retinoic acid inducible gene-like receptors in human tonsillar T lymphocytes. Immunology 133: 84–93. 21342182 10.1111/j.1365-2567.2011.03414.x PMC3088970

[27] Shaw MH, Reimer T, Sanchez-Valdepenas C, Warner N, Kim YG, et al (2009) T cell-intrinsic role of Nod2 in promoting type 1 immunity to Toxoplasma gondii. Nature immunology 10: 1267–1274. 19881508 10.1038/ni.1816 PMC2803073

[28] Caetano BC, Biswas A, Lima DS Jr, Benevides L, Mineo TW, et al (2011) Intrinsic expression of Nod2 in CD4+ T lymphocytes is not necessary for the development of cell-mediated immunity and host resistance to Toxoplasma gondii. European journal of immunology 41: 3627–3631. 22002196 10.1002/eji.201141876 PMC3241608

[29] Kim YG, Park JH, Shaw MH, Franchi L, Inohara N, et al (2008) The cytosolic sensors Nod1 and Nod2 are critical for bacterial recognition and host defense after exposure to Toll-like receptor ligands. Immunity 28: 246–257. 18261938 10.1016/j.immuni.2007.12.012

[30] Mercier BC, Ventre E, Fogeron ML, Debaud AL, Tomkowiak M, et al (2012) NOD1 cooperates with TLR2 to enhance T cell receptor-mediated activation in CD8 T cells. PLoS One 7: e42170. 22848741 10.1371/journal.pone.0042170 PMC3407091

[31] Wu HJ, Ivanov, II, Darce J, Hattori K, Shima T, et al (2010) Gut-residing segmented filamentous bacteria drive autoimmune arthritis via T helper 17 cells. Immunity 32: 815–827. 20620945 10.1016/j.immuni.2010.06.001 PMC2904693

[32] Ria F, Penitente R, De Santis M, Nicolo C, Di Sante G, et al (2008) Collagen-specific T-cell repertoire in blood and synovial fluid varies with disease activity in early rheumatoid arthritis. Arthritis research & therapy 10: R135. 19014626 10.1186/ar2553 PMC2656238

[33] Tuohy VK, Lu Z, Sobel RA, Laursen RA, Lees MB (1989) Identification of an encephalitogenic determinant of myelin proteolipid protein for SJL mice. Journal of immunology 142: 1523–1527. 2465343

[34] Ria F, Gallard A, Gabaglia CR, Guery JC, Sercarz EE, et al (2004) Selection of similar naive T cell repertoires but induction of distinct T cell responses by native and modified antigen. Journal of immunology 172: 3447–3453. 10.4049/jimmunol.172.6.3447 15004144

[35] Rolla S, Nicolo C, Malinarich S, Orsini M, Forni G, et al (2006) Distinct and non-overlapping T cell receptor repertoires expanded by DNA vaccination in wild-type and HER-2 transgenic BALB/c mice. Journal of immunology 177: 7626–7633. 10.4049/jimmunol.177.11.7626 17114432

[36] Pircher H, Michalopoulos EE, Iwamoto A, Ohashi PS, Baenziger J, et al (1987) Molecular analysis of the antigen receptor of virus-specific cytotoxic T cells and identification of a new V alpha family. European journal of immunology 17: 1843–1846. 2961577 10.1002/eji.1830171226

